# Terrestrial Positional Behavior of Wild *Pongo pygmaeus*

**DOI:** 10.1002/ajpa.70245

**Published:** 2026-04-06

**Authors:** Emily R. Orlikoff, Gene R. Estrada, Andrew J. Marshall, Heiko U. Wittmer, Endro Setiawan, Cheryl D. Knott, Lauren Sarringhaus, Laura M. MacLatchy

**Affiliations:** ^1^ Department of Anthropology University of Michigan Ann Arbor Michigan USA; ^2^ Department of Ecology and Evolutionary Biology University of Michigan Ann Arbor Michigan USA; ^3^ School of Environment and Sustainability University of Michigan Ann Arbor Michigan USA; ^4^ Program in the Environment University of Michigan Ann Arbor Michigan USA; ^5^ Program in Computing for the Arts and Sciences University of Michigan Ann Arbor Michigan USA; ^6^ Faculty of Biology and Agriculture Universitas Nasional Jakarta Indonesia; ^7^ School of Biological Sciences Victoria University of Wellington Wellington New Zealand; ^8^ Gunung Palung National Park Bureau Ketapang West Kalimantan Indonesia; ^9^ Department of Anthropology Boston University Boston Massachusetts USA; ^10^ Department of Biology Boston University Boston Massachusetts USA; ^11^ Department of Biology James Madison University Harrisonburg Virginia USA; ^12^ University of Michigan Museum of Paleontology Ann Arbor Michigan USA

**Keywords:** camera‐trap, *Pongo pygmaeus*, positional behavior, terrestrial, wild

## Abstract

**Objectives:**

As a predominantly arboreal animal in the wild, the terrestrial positional behavior of *Pongo pygmaeus* is poorly understood, having been studied almost exclusively in captive settings. This study uses camera‐trap footage to provide the first assessment of wild orangutan terrestrial locomotor and postural behavior on natural substrates.

**Materials and Methods:**

Video footage of orangutans from motion‐activated cameras in the Gunung Palung National Park, West Kalimantan, Indonesia was collected over a five‐year period. The resulting 100 instances of orangutan terrestriality were analyzed to document incidences of positional behavior, including hand and foot postures as well as overstride pattern during locomotion and qualitative assessment of hindlimb joint excursions during orthograde behaviors.

**Results:**

When locomoting terrestrially, *Pongo pygmaeus* primarily engaged in quadrupedal fist‐walking with heel‐strike. Wrist position and overstride pattern during quadrupedal walking were variable both within and between individuals. For posture, individuals were captured standing upright more often than pronograde, both monopedally and bipedally, and almost always with full extension of the hip and knee.

**Discussion:**

These observations of wild orangutan terrestrial positional behavior address prior ambiguities related to hand and foot positioning during locomotion. Maintained full extension of the hindlimb in the absence of substrate compliance indicates the form of bipedalism orangutans use in the trees translates to natural terrestrial substrates. Finally, the high proportion of observations with asymmetrical movement and posture may indicate a lateral decoupling of limbs in addition to the expected fore‐ and hindlimb independence of apes, suggesting orangutan positional adaptability is intrinsic regardless of substrate.

## 1 Introduction

Orangutans are the most arboreal of the great apes, and while their positional behavior (posture and locomotion) in the trees is well documented (e.g., Davenport 1967; Sugardjito and Van Hooff 1986; Cant 1987; Thorpe and Crompton 2006; Manduell et al. 2011), little is known about their postural and locomotor behavior when they come to the ground in the wild due to the rarity of their descents (Davenport 1967; Cant 1987; Crompton et al. 2010). The terrestrial movements of orangutans have been primarily studied in captive individuals living in predominantly terrestrial environments with limited access to arboreal substrates (e.g., Tuttle 1967; Susman 1974; Sarmiento 1985). This limitation has implications for our understanding of orangutan locomotion, as differences in habitat structure between captive and wild orangutans appear to influence both positional behavior and skeletal morphology (Sarmiento 1985; Casado et al. 2021; Kamaluddin et al. 2022). Thus, the locomotion and posture of wild orangutans when terrestrial is a critical gap in knowledge.

Here, we review findings of earlier work on orangutans, both captive and wild, in terms of their morphology and positional behavior compared to other great apes. We then present our analysis of 100 instances of orangutan terrestriality over 5 years using camera‐trap data collected at Gunung Palung National Park, West Kalimantan, Indonesia and address several ambiguities concerning the natural positional behavior of orangutans on the ground.

### 1.1 Orangutan Variation and Terrestriality

Wild orangutans are only found on the islands of Borneo and Sumatra. Sumatran orangutans are believed to be almost completely arboreal, rarely descending to the ground (Rijksen 1978; Thorpe and Crompton 2006), possibly due to the presence of terrestrial predators such as tigers ( *Panthera tigris* ; MacKinnon 1974; Goodrich et al. 2022). While terrestrial behavior in wild *Pongo pygmaeus* has been observed in Borneo, it is still rare despite the absence of nonhuman predators. It was speculated that Bornean adult male orangutans were the predominant age‐sex class to descend to the ground due to lower energetic costs of terrestrial travel and reduced predation risk (MacKinnon 1974; Crompton et al. 2010). However, while flanged adult males are the most commonly (and easily) identified age‐sex class on the ground at most research sites in Borneo (Ancrenaz et al. 2014; Ashbury et al. 2015; although see Loken et al. 2013), each camera‐trap study captured all age‐sex classes on the ground (Loken et al. 2013; Ancrenaz et al. 2014; Ashbury et al. 2015).

Observing orangutans in the wild is valuable, as developmental context has been shown to have behavioral and epigenetic morphological consequences (Sarmiento 1985; Casado et al. 2021). Familiarity with space and substrate availability in captivity may lead to more stereotypical movements than in the wild (Sarmiento 1985; Isler and Thorpe 2003; Casado et al. 2021; Kamaluddin et al. 2022). For example, Isler and Thorpe (2003) found that captive orangutans exhibit faster and longer steps during vertical climbing than do wild orangutans. This tendency has been linked to larger radiocarpal ligaments (inferred from attachment sites) in captive versus wild orangutans due to the need for enhanced wrist stabilization during such behaviors (Casado et al. 2021). Sarmiento (1985) identified several further distinctions between captive and wild‐caught skeletal remains, finding, for example, that captive orangutans (and African apes) exhibited higher humeral and femoral torsion than wild orangutans, and attributed this to more frequent propulsion in the anteroposterior rather than mediolateral plane in the former. Additional distinctions between captive and wild‐caught skeletal morphology in the wrist indicated a similarity in weight‐bearing in the forelimb between captive orangutans and African apes (Sarmiento 1985), potentially reflecting the increased terrestriality fostered by captivity (Kamaluddin et al. 2022). These and other observed differences in captive and wild orangutan skeletal morphology may be further illuminated by an enhanced understanding of their natural terrestrial positional behavior.

### 1.2 Great Ape Morphology and Positional Behavior

Understanding how great apes positionally engage with their natural environment is key to investigating the evolutionary underpinnings of their morphological adaptations. Apes are distinct from monkeys in their postcranial morphology, exhibiting longer forelimbs than hindlimbs, dorsally placed scapulae, a dorsostable lumbar column, strong manual and pedal grasping, and high postcranial joint mobility (e.g., Cartmill and Milton 1977; MacLatchy et al. 2021). The resulting orthogrady and fore‐ and hindlimb decoupling allow body weight to be distributed across multiple supports and so traverse arboreal substrates more effectively (MacLatchy et al. 2023). Despite these morphological similarities, extant great apes exhibit a range of arboreal and terrestrial modes of positional behavior (e.g., Hunt 2016) and live in a variety of habitats (e.g., Watts 2012). Recognizing commonalities and differences in great ape positional behavior across a range of environmental contexts may offer deeper insight into their adaptive evolution.

While all great apes navigate a complex canopy characterized by discontinuity and variable support orientation, size, and compliance, orangutans are the only predominantly arboreal taxon and have been found to engage in a wider range of positional behaviors than their African ape counterparts (Thorpe and Crompton 2006). Orangutan arboreal positional behavior is often referred to as “quadrumanous,” whereby the hands and feet utilize the same grasping mechanism (digits II–V used as a hook) and the extreme flexibility of the shoulder and hip allows them to grasp any substrate within reach of any of their limbs (Rose 1988). Povinelli and Cant (1995) describe orangutan locomotion as being highly adaptable with decreased stereotypy in limb movements to accommodate the high level of substrate compliance and complexity in the forests orangutans occupy. How such arboreal adaptability translates to terrestrial substrates remains unclear.

As part of their arboreal specialization, orangutans exhibit the highest intermembral index of the great apes (Schultz 1973), as well as a relatively short trunk (Pilbeam 2004; McCollum et al. 2010). These characteristics consequently render the torso more erect (often exceeding 45°) when engaging in terrestrial pronograde positional behavior. This tendency was first noted by Theodore Huxley (1894, 51‐52): “The very long arms…raise the body of the Orang remarkably, so that he assumes much the posture of a very old man bent down by age, and making his way along by the help of a stick”.

### 1.3 Great Ape Hand and Foot Postures

African apes spend more time on the ground than in the trees, with *Gorilla* species being more terrestrial than species of *Pan* (Tuttle and Watts 1985; Doran 1996; Hunt 2016). This variability in the use of terrestrial substrates underlies morphological differences among the great apes. As the part of the postcranium directly in contact with the substrate, the hands and feet have been argued to be highly diagnostic of positional behavior (Sarmiento and Marcus 2000) and consequently much studied in primates (e.g., Napier 1960, 1967; Tuttle 1967; Gebo 1985; Sarmiento 1985, 1988; Stern et al. 1995; Jungers et al. 1997; Sarmiento and Marcus 2000; Lemelin and Schmitt 2007; Patel et al. 2015; Schmitt et al. 2016; Kivell et al. 2016; Thompson et al. 2018; Zeininger et al. 2022; Prang 2024).

**FIGURE 1:**
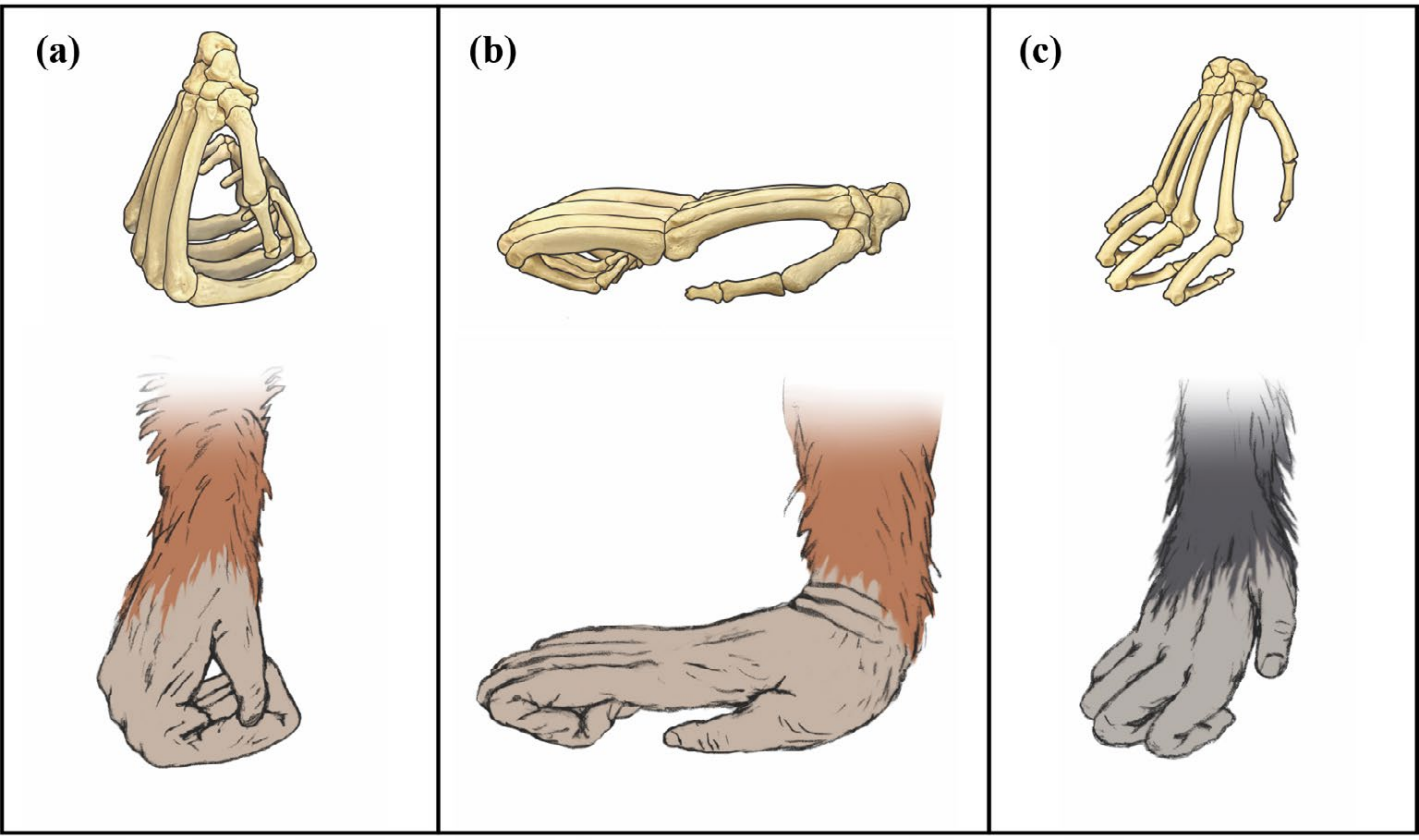
Representations of the skeletal anatomy in three hand postures (top row) with depictions of the hands of the apes below. (a) fist‐walking, whereby the dorsal surface of proximal phalanges of digits II–V contact the ground with the interphalangeal joints flexed (Tuttle 1967); (b) modified palmigrady, whereby the proximal interphalangeal joint is flexed in digits II–V and the dorsal aspect of the distal interphalangeal joint and distal phalanges contact the ground together with the palm of the hand (Sarmiento 1985); and (c) knuckle‐walking, whereby the dorsal surface of the intermediate phalanges of digits II–IV contact the ground (Tuttle 1967). Images are drawn from photographic and skeletal references of orangutans ([a] and [b]) and bonobos (c).

During terrestrial quadrupedal locomotion, African apes usually knuckle‐walk, a mode in which the dorsal surface of the intermediate phalanges of digits II–IV make contact with the ground (Tuttle 1967; see Figure 1c). In a focal‐follow study of 53 chimpanzees from Ngogo, Kibale Forest, Uganda, 100% of the terrestrial quadrupedal walking bouts (*N* = 1008) were found to be knuckle‐walking (Sarringhaus et al. 2022). However, this uniformity in hand usage is not universal across great apes. A video‐based study of wild mountain gorillas based on 503 captured instances of hand postures (*N* = 77 individuals, from Bwindi Impenetrable Forest in Uganda and Volcanoes National Park in Rwanda) found that while the majority of hand contacts during terrestrial quadrupedalism were knuckle‐walking, three other hand postures were also used (Thompson et al. 2018). This variation in mountain gorilla hand postures calls for further scrutiny in other great ape species.

Orangutans have been characterized as typically fist‐walking on the ground (see Figure 1a). Tuttle (1967) performed a comparative study of great ape hand anatomy and postures employed during quadrupedal walking in a laboratory setting. In his assessment of 26 captive orangutans, he identified five different hand postures, including three variations of fist‐walking (standard, modified, and abducted) and two variations of palmigrady (standard and modified) (see Figure 1b), distinguished by thumb contact and wrist position in the former and digit flexion in the latter. Subsequently, Tuttle and Beck (1972) reported use of knuckle‐walking hand postures during bipedal squatting and quadrupedal locomotion in a flanged male Bornean orangutan (Felix) at the Brookfield Zoo, Chicago, noting these occurred on a wet surface. Susman (1974) performed a follow‐up study of the same individual supporting their findings. Although these studies were of a single adult male, such behavior in captivity may not be exceptional, as we observed quadrupedal knuckle‐walking in an adult female (Batang) at the Smithsonian Zoo (Video S1), also on a smooth, wet surface. Observation of hand postures employed in the wild could thus inform our understanding of the repertoire used in the context of natural terrestrial substrates.

Orangutan hand and foot postures are affected by their highly derived cheiridia. Orangutans have the most curved proximal phalanges of any extant primate (Jungers et al. 1997) and the phalanges of the hands and feet in orangutans comprise a larger proportion of the total hand or foot length compared to those of the African apes (Zihlman and Underwood 2019). The morphological distinctions between orangutans and the African apes have promoted debate in the comparability of their performance of certain behaviors, particularly heel‐strike.

Heel‐strike plantigrady, in which the heel contacts the substrate first when walking, has long been suggested to be a defining trait of humans and great apes (Weidenreich 1923; Elftman and Manter 1935; Gebo 1992, 1993; Meldrum 1993; Schmitt and Larson 1995; Zeininger et al. 2020), although some exclude orangutans from this behavior. Gebo (1992) observed heel‐strike to occur only in African apes and humans and proposed it to be an essential preadaptation for hominin bipedalism. In the case of orangutans, he reported that during touchdown, the entire lateral foot of the orangutan makes contact with the ground rather than the heel first, and therefore did not view this weight transfer as true heel‐strike. However, this finding was disputed (Meldrum 1993), and Schmitt and Larson (1995) made the distinction that since the lateral aspect of the heel touches the ground before the rest of the foot in *Pongo pygmaeus* , the placement should be considered a variation of heel‐strike.

There is preliminary evidence of captive orangutans occasionally engaging in heel‐strike plantigrady during bipedal locomotion (see Crompton et al. 2003, 2008). Bipedal orangutans walking across force plates are reported to produce vertical ground reaction force curves with a distinct peak at heel contact in 25% of cases (Crompton et al. 2008). While peak plantar pressure maps demonstrate that orangutans do tend to load the lateral aspect of their foot during bipedal walking (Crompton et al. 2008), this pattern may not be unique among great apes as bonobos produce similar plantar pressure distributions (Vereecke et al. 2003; Crompton et al. 2008). Accordingly, Vereecke et al. (2003) suggested that the term “inverted heel‐strike plantigrady” be used to characterize the lateral pressure observed in great apes to distinguish it from the “full‐contact heel‐strike plantigrady” observed in humans. Thus, some findings suggest that heel‐strike performance in captive orangutans may merit inclusion with the African apes. However, given that orangutan terrestrial locomotion has been mainly studied in zoo and laboratory settings on artificial, flat surfaces such as concrete, it remains unclear how they engage in locomotion on the ground in the wild. Navigation of a natural forest floor, with its uneven surfaces, living and decaying vegetation, and variable inclination, may influence both hand and foot touchdown performance.

### 1.4 Great Ape Quadrupedalism

Observation of wild orangutan terrestrial quadrupedalism affords further insight into recognized behavioral characteristics of great apes. In particular, overstriding during quadrupedal locomotion is a distinct behavior of primates, whereby the foot passes the ipsilateral hand at touchdown (Hildebrand 1967; Reynolds 1985; Larson 1998). However, great apes have been shown to exhibit their own variation of this behavior. Due to their relatively long arms and short legs, great apes walk with their torso oblique to the direction of travel, creating an asymmetry in hand and foot placement such that one foot is placed medial to its ipsilateral hand (i.e., the inside foot) and the other lateral to its ipsilateral hand (i.e., the outside foot; Huxley 1894; Hildebrand 1967; Reynolds 1985). Although this tendency is recognized in all great apes, few comparative studies have been conducted to appreciate potential taxonomic distinctions in its execution. Previous studies reporting overstride performance in great apes, while limited, have found variation between taxa in relative force production between inside and outside limbs (Demes et al. 1994) as well as in foot preference between individuals (D'Août et al. 2004). Despite documented variation in great ape overstride behavior, it has yet to be assessed in a wild setting.

### 1.5 Great Ape Bipedalism

The positional behavior and ecology of great apes have also been extensively studied in the search for anatomical and physiological traits, as well as ecological circumstances, that may be relevant for the adoption of terrestrial bipedality in the hominin lineage (Rodman and McHenry 1980; Richmond et al. 2001; D'Août et al. 2004; Sockol et al. 2007; Pontzer et al. 2009; Thorpe, Holder, and Crompton 2007; O'Neill et al. 2015; Drummond‐Clarke et al. 2022; Sarringhaus et al. 2024). While African apes, particularly chimpanzees, have been the primary focus for such research given their closer phylogenetic relationship to humans (Ruvolo et al. 1991), orangutans exhibit positional behaviors in the trees that have been argued to provide a better model for incipient bipedality (Crompton et al. 2003, 2008, 2010; Thorpe et al. 2014).

Bipedalism is used occasionally by all apes (Nowak and Reichard 2016), but the form practiced by orangutans is unique among the apes in that it is performed with fully extended hips and knees, as in humans (Crompton et al. 2010). Orangutans use bipedal locomotion approximately 7% of the time in the trees (Thorpe and Crompton 2006), and while this mode is relatively rare, it has been argued to be critical to utilizing compliant, peripheral branches in tree crowns both to forage and to travel between trees (Thorpe, Holder, and Crompton 2007). Access to such substrates is argued to be necessary to avoid descending to the ground, saving energetic costs and limiting predation risk (Crompton et al. 2010). This hypothesis is supported by the observation that nearly 60% of their arboreal locomotion occurs on supports that are less than 10 cm in diameter, a size likely to bend under their body weight (Thorpe, Crompton, and Alexander 2007). However, their employment of such postures terrestrially, where compliance does not pose the same constraints, is poorly understood in the wild.

### 1.6 Goals of the Present Study

The present study is the first to examine terrestrial positional behavior of wild orangutans ( *Pongo pygmaeus* ) on natural substrates. Studying primate positional behavior in nature is important as the complexity, availability, and mechanical properties of substrates vary between wild and captive settings, influencing performance (Isler and Thorpe 2003). Wild primate locomotion is much more heterogeneous compared to that in laboratory and zoo settings (Hosey 2005; Kamaluddin et al. 2022), and this complexity cannot be replicated by even the most naturalistic captive studies. Critically, studies in the wild also document the ecological conditions under which behaviors naturally occur.

Camera‐trap footage affords a powerful means of assessing the natural terrestrial behavior of orangutans up‐close, while avoiding the potential influence of researcher presence. In this study, we present an analysis of 100 instances of wild orangutan terrestriality collected over 5 years at Gunung Palung National Park, West Kalimantan, Indonesia. The frame‐by‐frame analysis of this footage enables the assessment of several enduring questions concerning orangutan terrestrial behavior in the wild. Here, we report the age‐sex classes as well as the behavioral context of individuals observed on the ground and specifically address the following questions:

1. What positional behavioral modes and submodes are employed by terrestrial orangutans in the wild? This includes the investigation of hindlimb joint excursions during orthograde behaviors.
2. When quadrupedal walking on the ground in the wild, what hand postures are employed and what are the characteristics of their overstride pattern?
3. When walking on the ground, both quadrupedally and bipedally, do orangutans exhibit heel‐strike in the wild?

Our findings provide an initial opportunity to compare the terrestrial positional behavior of wild orangutans with that of the well‐studied African apes and consider the wider implications for hominoid evolution. We also examine the pertinence of terrestrial orangutan observations for the origins of bipedalism in the hominin lineage as well as the effects of their quadrumanous arboreal adaptations on their terrestrial behavior.

## 2 Materials and Methods

### 2.1 Study Site

**FIGURE 2:**
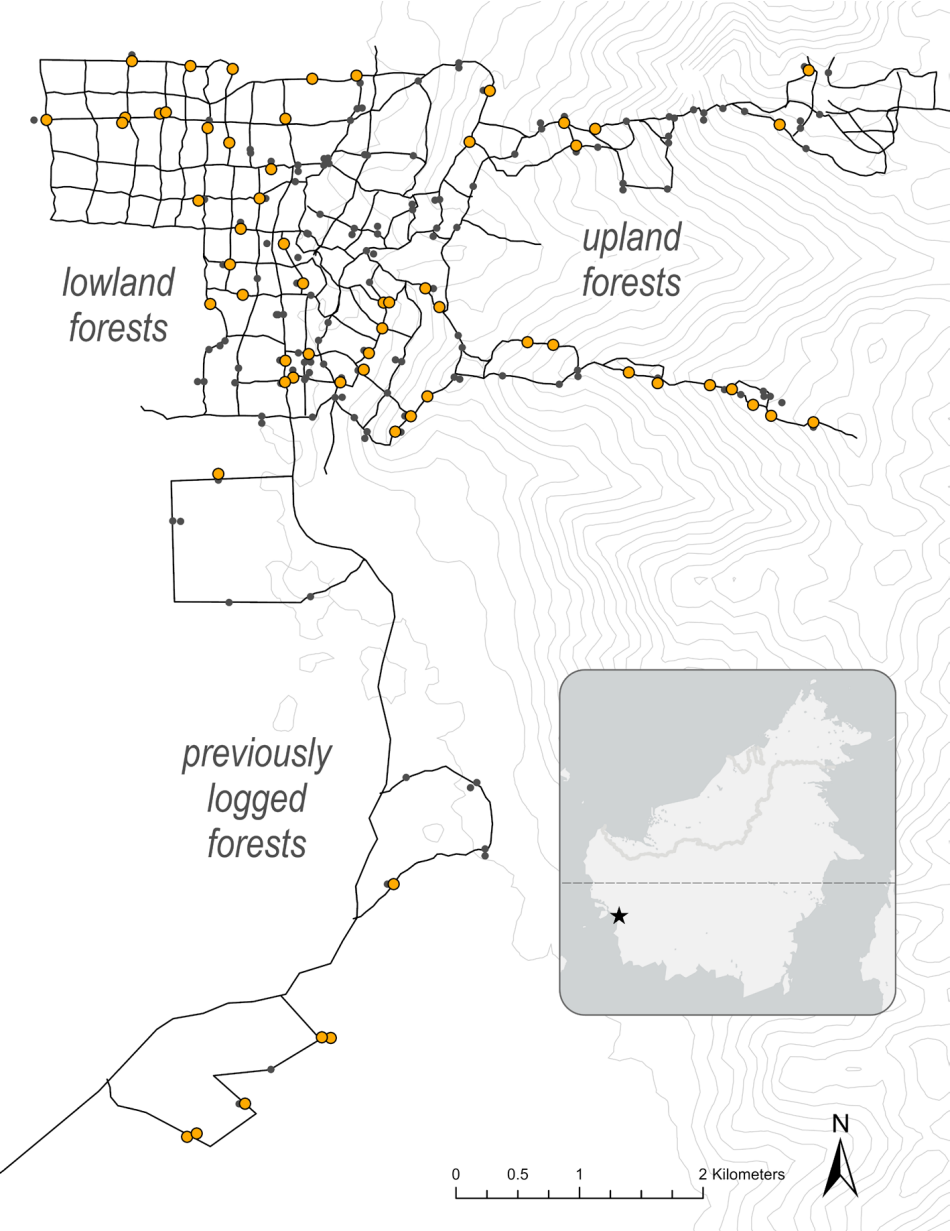
Map of study site. Black lines depict the trail system at Cabang Panti Research Station. Black points represent camera‐trap placements. Orange highlighted points represent cameras that have captured orangutan sightings. Upland forests include the upland granite and montane forests (350–800 m above sea level (asl) and 750–1100 m asl, respectively). Lowland forests include peat and freshwater swamps (0–5 m asl and 5–10 m asl, respectively), alluvial bench forests (5–100 m asl), lowland sandstone forests (20–200 m asl) and lowland granite forests (200–400 m asl). The star in the inset map marks the location of the site in Borneo.

The camera‐trap data in this study was collected at the Cabang Panti Research Station (CPRS), a tropical rainforest site located in Gunung Palung National Park, West Kalimantan, Indonesia (1°13′ S, 110°7′ E; Figure 2). The trails at CPRS access seven unique and contiguous forest types over a 34 km^2^ area and traverse an elevational gradient of over 1100 m. Forest types at CPRS differ in elevation, plant species composition, plant phenology, and forest structure (Marshall et al. 2021) and include the following: both peat and freshwater swamp forests, alluvial bench forests near rivers, lowland sandstone forests, montane forests, and both lowland and upland granite forests. Directly south of the main trail system at CPRS is a previously logged area of alluvial bench forest, called the Rangkong River research area (see Figure 2).

The orangutan population within Gunung Palung National Park has been studied since 1994 (Knott et al. 2021) and has been estimated to be approximately 2500 individuals (Johnson et al. 2005) with an average density of 3.0 individuals/km^2^ (Johnson et al. 2005; Marshall et al. 2014). Orangutan abundance at CPRS has been shown to vary widely across time and space, with each forest type experiencing periods of low and high abundance according to food availability (Marshall et al. 2021).

### 2.2 Camera‐Trap Data Collection

Motion‐activated cameras collected footage of orangutans ( *Pongo pygmaeus* ) from Gunung Palung National Park from June 2015 to September 2020. At any given time, there were 35–72 active cameras rotating between 207 locations across the seven forest types. Ultimately, this amounted to 53,311 camera‐trap days with 36,356 videos containing fauna. Out of the 69 species recorded, orangutans ranked as the 24th most commonly captured taxon, yielding 120 video files of orangutan observations. Videos where no data could be collected (i.e., age, sex, and behavior indiscernible) were excluded from this study. The motion‐triggered cameras automatically recorded for 20 s with a 10 s pause before recording again. The frame rate for the cameras ranged from 15 to 30 frames per second. Most cameras were placed on forest trails to ensure visibility of fauna (see Figure 2), although a minority were placed off‐trails to permit assessment of trail placement on capture frequencies. Quantitative kinematic assessment of joint and segment angles was not feasible due to uneven and obstructed terrain and high variation in body orientation relative to the camera.

For each video, age category (adult and subadult) and sex (male with flanging noted, female, and unknown) of the individuals present was documented. Subadults were not categorized by sex and were distinguished from adults by their accompaniment with an adult female (i.e., their mother) as well as their relative size. Infants were also documented when present in a video but, as they were carried by their mother (distinguishing them from the subadult sample), were not included otherwise in the study. Additionally, behavioral context was examined using the categories travel, vigilance, feed, rest, and play (Table 1).

**TABLE 1:** Ethogram for behavioral context of descent.

Behavior	Definition
Travel	Actively moving to a new location
Vigilance	Not moving and scanning surroundings
Feed	Actively bringing food to mouth
Rest	Not moving and not actively focused on surroundings
Play	Play wrestling/fighting (social)

All positional behavioral modes and submodes engaged in by individuals on the ground were coded (Tables 2 and 3). Repeated observations (camera re‐activated) of the same individual were collapsed into a single bout of positional behavior. For example, if there were multiple videos of an individual sitting in view of the camera in the same place, this was considered a single observation of “sit”. Should the individual subsequently change their positional behavior, such as transitioning from sitting to quadrupedal walking, this would be included as part of the same terrestrial instance.

**TABLE 2:** Definitions of locomotor behavior modes (in bold) and submodes (*in italics*) observed in the footage.

Mode *submode*	Definition
**Bipedal walk**	The hindlimbs provide support and propulsion, with only insignificant contributions from other body parts.^a^ In this study, a minimum of one step was required, which we define as the hindlimb contacting the ground in a deliberate, controlled movement.
*Extended bipedal walk*	Bipedal walk in which the hip and knee are [nearly] extended [in the standing leg].^a^
*Hand‐assisted Extended bipedal walk*	Bipedal walk in which hindlimbs bear more than 50% of body mass and hip and knee are in full extension. One or both forelimbs are used to assist, either in suspension or compression, and bear more than their own weight.^b^
**Quadrupedal walk**	Locomotion on supports angled at < 45°. Typically, all the four limbs contact the support in a particular sequence. The torso is pronograde or roughly parallel to the support. Walking is distinguished from running principally by its slow or medium speed.^a^
*Symmetrical gait walk*	Diagonal sequence, diagonal couplets gait. Limbs are extended. Symmetrical gaits are characteristic of most primate walking.^a^
*Crutch walk*	Quadrupedal walk in which the forelimbs move forward simultaneously with fully extended elbows. After the hands are planted, the body and hindlimbs are swung through the forelimbs.^a^
**Forelimb‐hindlimb swing**	Suspensory locomotion…utilizing both forelimbs and hindlimbs in both orthograde and pronograde positions.^b^
*Ipsilateral swing*	Swinging from ipsilateral fore‐ and hindlimb.^b^
**Somersault**	A forward movement in which the superior surface of the head initially contacts the substrate and bears the weight of the body followed by the posterior surface of the head, neck, and back as the body does a complete revolution before the plantar surfaces of the feet contacts the substrate.^c^

^a^ As described in Hunt et al. (1996).

^b^ As described in Thorpe and Crompton (2006).

^c^ As described in Sarringhaus et al. (2014).

**TABLE 3:** Definitions of postural behavior modes (in bold) and submodes (*in italics*) observed in the footage.

Mode *submode*	Definition
**Orthograde stand**	More than half the body weight is supported by one or two hindlimb(s) in compression.^a^
*Extended bipedal stand*	Hip and knee are completely extended^b^, but there is no significant support from the forelimb(s).^c^
*Extended bipedal stand/Forelimb‐suspend*	More than half of the body weight is supported by the hindlimbs, with the hips and knees completely extended. However, there is significant support from a suspended forelimb.^c^
*Extended monopedal stand*	Body mass is supported by standing on one leg, with insignificant contributions from other body parts. Hip and knee are completely extended.^d^
*Extended monopedal stand/Forelimb‐suspend*	As in “[Extended] Bipedal Stand/Forelimb‐Suspend”, but with only one hindlimb bearing weight.^d^
*Extended monopedal stand/Forelimb compression*	As in “[Extended] Monopedal Stand” but with one or both arms below the shoulder and supporting body weight in compression.^d^
*Flexed bipedal stand*	Standing on the hindlimbs with no significant support from any other body part. The torso is typically held at an approximately 45°angle. The hips and knees are flexed.^c^
*Flexed bipedal stand/Forelimb‐suspend*	More than half of the body weight is supported by the hindlimbs, with flexed hips and knees. There is significant support from a forelimb oriented in a forelimb‐suspend pattern.^c^
*Flexed bipedal stand/Forelimb‐assisted*	As in “Flexed Bipedal Stand” but with assistance from one or both forelimbs which do not support more than their own weight.
**Pronograde stand**	Three or four limbs bear the majority of the body weight on a horizontal or near horizontal substrate. Limbs can be flexed or extended.^a^
*Quadrupedal stand*	Four‐limbed standing on horizontal or subhorizontal supports.^c^
*Tripedal stand*	As above except with two hindlimbs and one forelimb bearing weight.^c^
**Sit**	The ischia bear a substantial portion (usually more than half) of the body weight; the torso is relatively orthograde.^c^

^a^ Modified from Sarringhaus et al. (2014).

^b^ Extension in the hip and knee is evaluated anteroposteriorly (abduction in hip still possible).

^c^ As described in Hunt et al. (1996).

^d^ As described in Thorpe and Crompton (2006).

Orthograde and pronograde stands were distinguished according to whether the forelimb(s) supported weight on the ground rather than by torso angle (see Table 3). The protocol developed by Hunt et al. (1996) for determining weight‐bearing limbs (i.e., supporting more than its own weight) in wild settings was applied in classifying positional behavioral modes and submodes. Joint excursions in the hip and knee were evaluated according to the relative positions of body segments in the anteroposterior plane only. Full extension (~180° between body segments) can thus occur whether the hip is abducted or adducted (i.e., in the mediolateral plane) and/or the knee alignment is varus or valgus.

During locomotor bouts, hand posture (Figure 1), foot posture at the touchdown phase of the gait cycle, presence or absence of toe flexion (i.e., curling of digits II–V), and overstride pattern were examined when discernible. Hand posture was analyzed during quadrupedal walking bouts with each visible hand contact coded as an individual observation. Hand posture during pronograde stands was not included. Given the variation in the hand and wrist observed during fist‐walking by Tuttle (1967), wrist position was also considered and was categorized as extended (< 180°; see Figure 1b), neutral (~180°; see Figure 1a,c), or flexed (> 180°). Thumb contact was not considered.

In cases where the foot posture at initial contact could be seen, the type of plantigrade contact was coded as either “heel‐strike” or “full‐foot.” Heel‐strike is defined by the heel making initial contact with the ground at touchdown followed by the forefoot. In full‐foot contact, the entire plantar surface of the foot contacts the ground simultaneously. Foot posture was examined during both quadrupedal and bipedal walking bouts. Each visible foot contact was coded as an individual observation. Individuals moving on inclined terrain were excluded from foot contact analysis due to enhanced dorsiflexion (upward progression) and plantarflexion (downward progression) of the ankle.

Coding of behavior was tested for interobserver reliability to ensure accuracy of data. Positional behavioral modes (locomotion *N* = 77, posture *N* = 50) were coded by both EO and LS without discrepancy (*N* = 127/127). Independent coding of hand postures and heel‐strike (*N* = 86 total) was also conducted for two‐thirds of the dataset (*N* = 58) by EO and LS with 95% agreement. There were three instances of differences in the coding of the hands and feet between the two observers. Two instances were discussed and resolved, and the third was excluded from the analysis.

The overstride pattern was also analyzed for quadrupedal walking bouts. In cases in which the placement of the left or right foot was maintained as the inside foot, the overstride pattern was considered to be “consistent.” When the individual altered the inside foot, the overstride pattern was considered to be “mixed.” Overstride pattern was only analyzed for bouts with two or more gait cycles.

### 2.3 Data Analysis

Within the categories of positional behavior, hand posture, foot posture at initial contact, toe flexion, and overstride pattern, the counts of each behavioral variation were compared to the total counts within each category. Age, sex, and context of terrestriality were also documented. Statistical comparisons of frequencies between age and sex were not included. Within each category of coded behavior, the number of observations for each age or sex class was too small to conduct statistical tests. However, whether behaviors were performed by adults of both sexes, or by both adults and subadults, is reported below to shed light on any potential variation. As this is the first documentation of the natural positional behaviors of wild orangutans on the ground, this study is meant to serve as a foundational dataset upon which to build.

## 3 Results

Of the 100 instances of terrestrial orangutans in our sample, 77 involved a single individual and 23 captured two individuals, for a total of 122 individuals observed (Table 4). There was a single case where a second recording captured a female joining a male sitting, the latter whom we did not count twice. Dyads were predominantly mother and offspring (*N* = 18), but there were rare incidences of adult females traveling with adult males, both flanged (*N* = 2) and unflanged (*N* = 2). These females appeared to be young based on relative size. There was a single observation of two subadults, perhaps of similar age given their comparable body sizes, wrestling on the ground.

**TABLE 4:** Age and sex distributions for observed terrestrial orangutans.

Variable	Group	Sample size
Age	Adults	88
	Subadults	14
	Infants	8
	Unknown age	12
	Total	122
Sex (adults only)	Male—flanged	23
	Male—unflanged	8
	Male—flanging unknown	6
	Female	31
	Unknown sex	20
	Total	88

Behavioral context was most commonly travel (*N* = 67), followed by vigilance (*N* = 27), feed (*N* = 7), rest (*N* = 4), and play (*N* = 2). These behaviors were not mutually exclusive and are expected to be biased toward movement given the activation of the camera.

Seventy‐seven bouts of locomotion were observed (Table 5) and were overwhelmingly quadrupedal walking. Quadrupedal walking bouts mainly exhibited diagonal sequence gaits (submode symmetrical gait walk) apart from three instances of crutch walking. Bipedal walking was rare, but distributed across multiple submodes, including hand‐assisted extended bipedal walk, extended bipedal walk, and hand‐assisted flexed bipedal walk. Quadrupedal and bipedal walking were observed in both sexes and age groups. Crutch walking was only observed in adults. The single ipsilateral swing was performed by an adult female and the single somersault was performed by a subadult.

**TABLE 5:** Counts of positional behavioral modes and submodes.

Mode	Submode	Count
Locomotion (*N* = 77)		
Quadrupedal walking (*N* = 67)	Symmetrical gait walk	64
	Crutch Walking	3
Bipedal walking (*N* = 8)	Extended bipedal walk, forelimbs obscured	3
	Hand‐assisted extended bipedal walk	3
	Extended bipedal walk	1
	Hand‐assisted flexed bipedal walk	1
Forelimb‐hindlimb swing	Ipsilateral Swing	1
Somersault		1
Posture (*N* = 50)		
Orthograde stand (*N* = 21)	Extended monopedal stand/forelimb‐suspend	9
	Flexed bipedal stand/forelimb‐assisted	3
	Extended bipedal stand	2
	Extended monopedal stand, forelimbs obscured	2
	Extended monopedal stand	1
	Extended monopedal stand/forelimb‐compression	1
	Extended bipedal stand/forelimb‐suspend	1
	Flexed bipedal stand	1
	Flexed bipedal stand/forelimb‐suspend	1
Pronograde stand (*N* = 19)	Quadrupedal stand	15
	Tripedal stand	4
Sit	Sit	10

**FIGURE 3:**
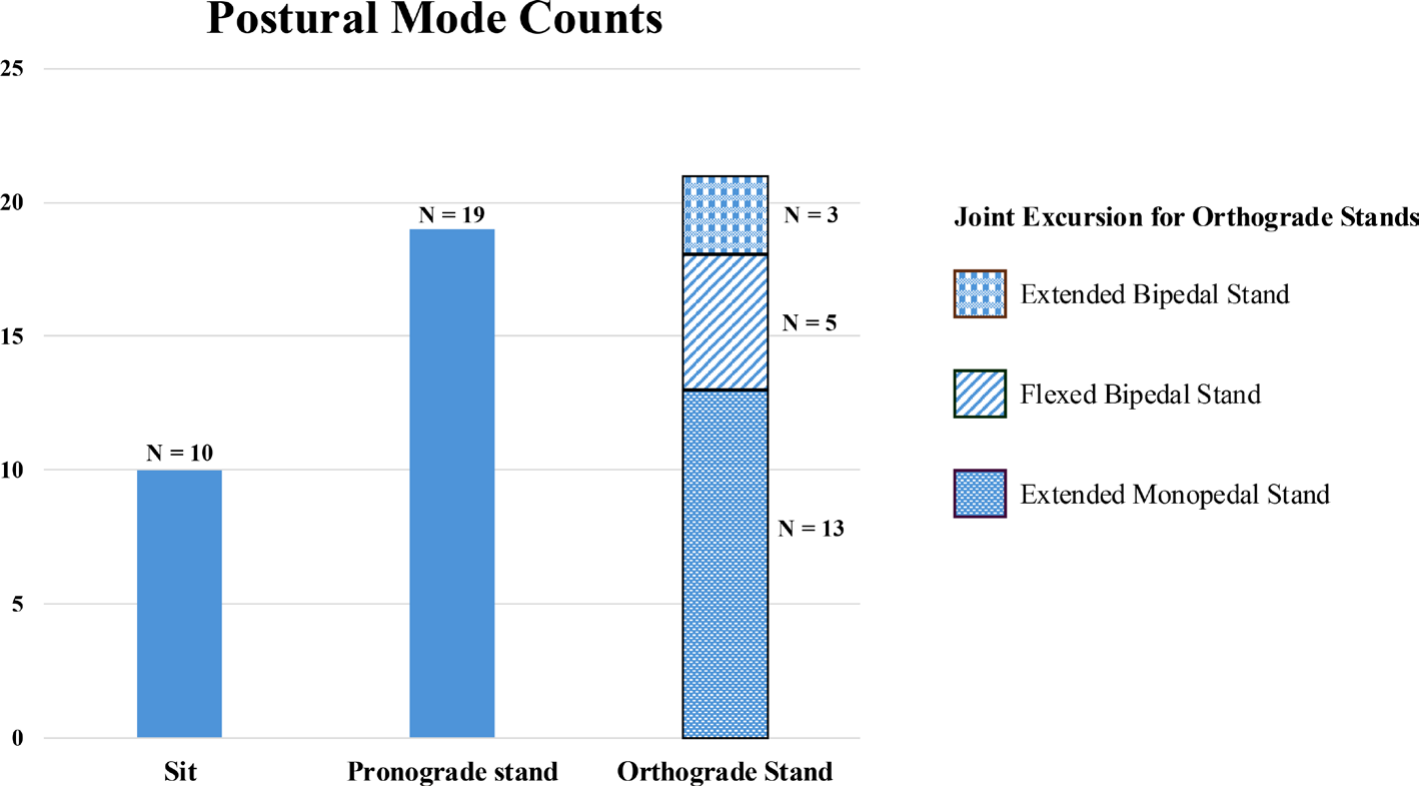
Counts of postural modes observed (*N* = 50). Variation in orthograde stands based on number of weight‐bearing legs (monopedal vs. bipedal) and hindlimb joint excursion (extended vs. flexed) are separated by pattern. Orthograde stand submodes (account for forelimb variation in addition to hindlimbs) are reported in Table 5.

The 50 bouts of posture observed (Figure 3; Table 5) fell into three primary modes including orthograde stand, pronograde stand, and sit. Pronograde stands were both quadrupedal and tripedal. Orthograde stands were distributed across nine submodes, with extended monopedal stand/forelimb‐suspend being most common. All postural modes, including orthograde stands, were observed in both sexes and age groups.

There were 49 occurrences of observable hand posture during 22 bouts of quadrupedal walking, performed by individuals of both sexes and age groups. Fist‐walking was the most frequently identified hand posture (*N* = 43) with the remainder being modified palmigrady (*N* = 6). Hand and wrist posture were coded separately for individual touchdowns as a few individuals employed different hand and/or wrist postures for each hand. In the 43 observations of fist‐walking, the wrist was coded as extended (*N* = 19) and neutral (*N* = 23), with one instance where the wrist position could not be determined.

Thirty‐seven foot touchdowns were observed during quadrupedal locomotion. All but three of these exhibited heel‐strike plantigrady. One subadult (*N* = 2) and one adult female (*N* = 1) did not exhibit heel‐strike during quadrupedal walking and instead used full‐foot contact. The adult female exhibited heel‐strike in other observed steps (*N* = 2), but the subadult did not and was the only subadult in the foot touchdown sample for quadrupedal walking. Of the three bipedal steps, full‐foot contact, but not heel‐strike, was observed. Two bipedal touchdowns were performed by a single flanged adult male and one by a subadult. The toes were curled during all instances of quadrupedal and bipedal walking when visible.

In the 15 bouts of quadrupedal locomotion where two or more gait cycles were observed for determination of the overstride pattern, seven were consistent (i.e., the individual maintained the same inside foot across gait cycles) while the remaining eight were mixed. Both sexes exhibited both patterns and the one subadult in this sample exhibited a consistent overstride pattern. There did not appear to be preference for either foot being inside or outside across the sample as a whole. For the individual footfalls, the left foot was coded as outside 19 times and inside 19 times and the right foot was coded as outside 21 times and inside 24 times.

## 4 Discussion

### 4.1 Demographics and Behavioral Context

This study documented orangutans of both sexes and age classes on the ground, supporting findings in previous studies for Bornean orangutans (Loken et al. 2013; Ancrenaz et al. 2014; Ashbury et al. 2015). The behavioral context observed for the terrestrial orangutans was predominantly travel, most often quadrupedally, but also bipedally. When standing (orthograde and pronograde), orangutans were most often observed in the context of vigilance, which was distinguished from rest alone by the individual actively scanning their surroundings. Feeding bouts were observed both sitting and standing, and both bipedally and monopedally when orthograde. As noted, the camera‐trap method biases context toward travel. However, the influence of context on behavioral performance in the wild may reflect selective factors for specific positional behaviors and will be worth further study once larger samples are available. For example, in a recent study of wild chimpanzees, a greater variety of behavioral contexts were associated with terrestrial bipedalism compared to arboreal bipedalism (Sarringhaus et al. 2024).

### 4.2 Positional Behavior

#### 4.2.1 Locomotor Behavior

Overstride during quadrupedal walking was relatively evenly split between mixed and consistent in pattern. While examination of this behavior is limited in the African apes, particularly in the wild, the observations in this study contrast with what has been observed in captive bonobos, who exhibit a preferred inside foot during quadrupedal walking (D'Août et al. 2004). The inside foot, being closer to the midline and thus the center of gravity, experiences a higher load than does the outside foot (D'Août et al. 2004), suggesting the more terrestrial African apes might be expected to have a “dominant” leg, but orangutans may not. Future studies will be needed to better evaluate this behavior across the great apes.

Our data also show that Bornean orangutans occasionally engage in bipedal walking when on the ground, accounting for 10.4% of the locomotor sample in this study. Bipedal bouts exhibit relatively extended hips and knees in the support leg. Although joint angles could not be effectively measured, qualitative assessment indicates that the extended hip and knee bipedalism observed in *Pongo* in the trees (Crompton et al. 2010) is maintained on the ground even in the absence of compliant substrates (see Videos S2). This contrasts with the bent‐hip, bent‐knee pattern of bipedalism observed in the African apes, particularly chimpanzees (e.g., see figure 1 in Sarringhaus et al. 2024 showing bent hip and knee joints during both arboreal and terrestrial bipedalism in chimpanzees) and supports previous studies documenting extended hindlimbs in bipedal captive orangutans (Tuttle and Cortright 1988; Crompton et al. 2003, 2008; Watson et al. 2009).

Crutch walking was observed three times in adults moving on relatively flat terrain (see Video S3). While the sample size is low, it is possible that this form of quadrupedalism may differ in some respects from that practiced by chimpanzees, who not only perform this behavior very rarely but tend to do so on downward slopes (Hunt 1992). Crutch walking in orangutans may be influenced by their relatively long arms, which shift weight toward the hindlimbs, facilitating the performance of this behavior. Data on the frequency of crutch walking in gorillas has not been published as far as we are aware.

#### 4.2.2 Postural Behavior

**FIGURE 4:**
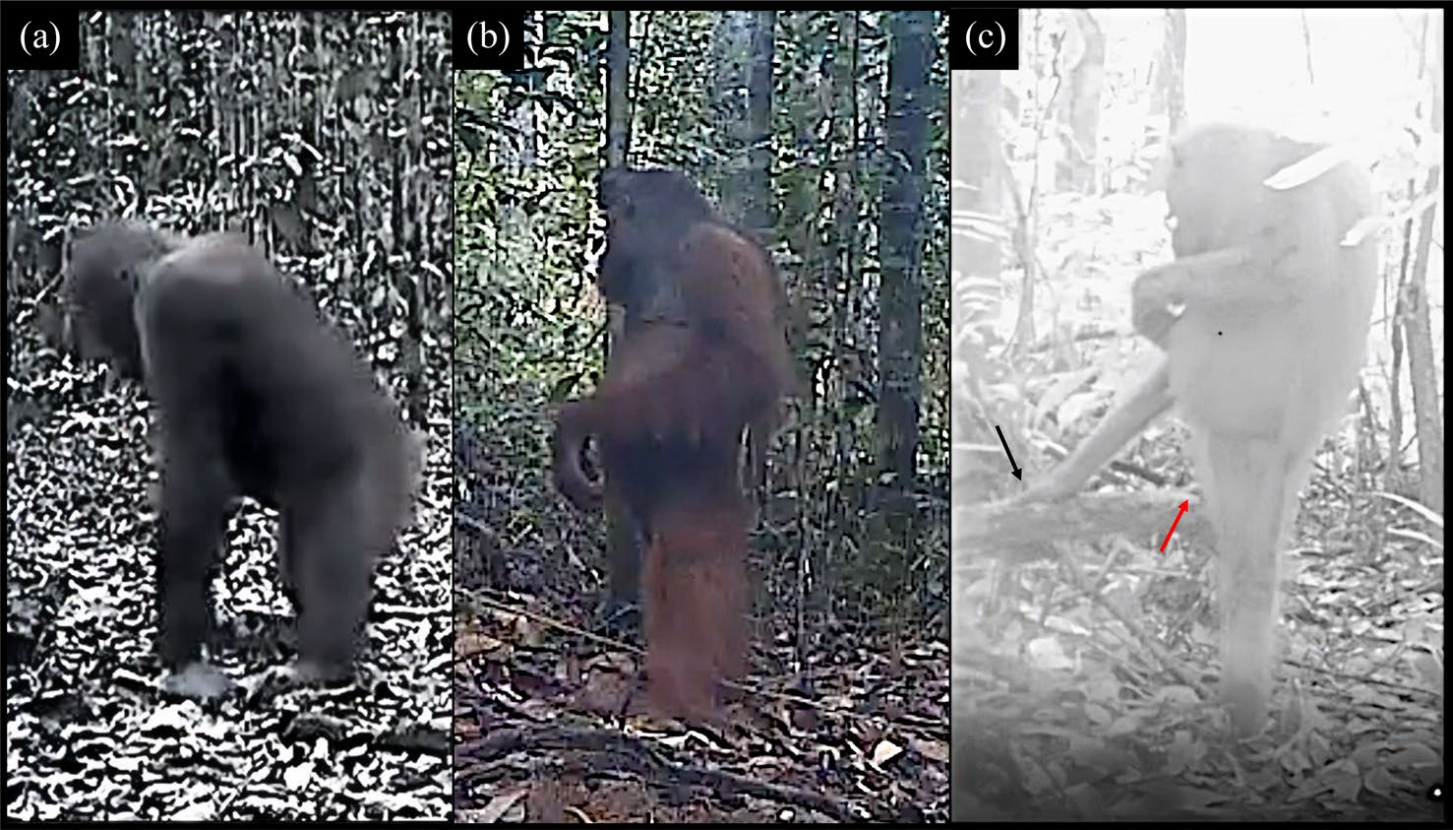
Orangutan terrestrial stands showing (a) quadrupedal, (b) bipedal, and (c) monopedal variations (black arrow pointing to right hand and red arrow pointing to right foot (only toe is visible in the image) resting on a horizontal log). Videos of (b) and (c) are available to view (Videos S4 and S5, respectively).

The orangutans in this study were observed standing fully orthograde, with most body weight borne on one or two hindlimbs, more often than standing pronograde (Table 5; Figures 3 and 4). This finding follows what has been reported for their postural behavior in the trees, where upright torsos are most common (Thorpe and Crompton 2006). As was the case in locomotion, orthograde standing was typically characterized by hindlimb extension, accounting for 76% of the sample (see Videos [Link], [Link]). While all monopedal orthograde stands exhibited a fully extended hip and knee in the weight‐bearing leg, bipedal stands were more variable in hindlimb joint excursions, with only three of the eight bipedal stand observations classified as extended. Of the five flexed bipedal stands, three fell into the submode flexed bipedal stand/forelimb‐assisted.

When standing monopedally, the other leg and/or arm(s) consistently held on to or rested on a substrate, such as a branch, liana, sapling, or trunk (see Figure 4c and Videos [Link], [Link], and S8). It thus seems that the differential limb use that characterizes orangutans in the trees, with limbs often oriented in different directions and grasping different substrates, persists on the ground. Such limb decoupling, even on terrestrial substates, indicates how intrinsic this tendency may be in orangutans.

### 4.3 Hand Posture

No knuckle‐walking was observed in *Pongo pygmaeus* in this study. Individuals primarily engaged in fist‐walking with a small proportion of modified palmigrady. Such variation in hand postures during quadrupedalism has been found in gorillas as well, with non‐knuckle‐walking hand postures correlating with substrate, pathology, and apparent individual preference (Thompson et al. 2018).

Both extended and neutral wrist positions were observed during fist‐walking, with orangutans using extended wrist postures nearly as often as neutral. Given that the extended wrist fist‐walking and modified palmigrady hand postures are distinguished by the flexion or extension of the metacarpophalangeal joints of digits II–V (see Figure 1), hand postures in orangutans may represent a fluid spectrum of use rather than stereotypic execution of one dominant position. In addition, there were multiple cases of asymmetry in hand and wrist posture employment, with two instances of individuals employing a fist for one hand and modified palmigrady for the other and two instances of fist‐walking with one wrist extended and the other neutral in position. Further investigation is warranted to assess the extent of hand use plasticity in the wild in orangutans, as well as in African apes, particularly in light of the findings by Thompson et al. (2018).

### 4.4 Heel‐Strike

The performance of heel‐strike when quadrupedal walking is consistent with the “inverted heel‐strike plantigrady” contact pattern described for the African apes by Vereecke et al. (2003). However, although the toes were invariably flexed when visible, foot posture and orientation were not consistent across individuals during the stance phase of the gait cycle in quadrupedal walking. Some individuals exhibited a highly inverted foot that was oriented anteroposteriorly prior to toe‐off (see Video S10) while others exhibited a less inverted foot that was more laterally oriented with a medially extended hallux (see adult female in Video S11). The flexed toes are therefore variable in their contact with the substrate according to the degree of inversion of the foot. This range in foot posture during quadrupedal walking may indicate considerable variation in plantar pressure distributions as has been reported in bonobos (Vereecke et al. 2003). As with hand posture, variation in foot posture in terrestrial orangutans may not lend itself to placement in the discrete categories used for taxa that are frequently quadrupedal.

Such variable observations of heel‐strike presented here may indicate its performance should not be used as a character state when making phylogenetic inferences. The performance of heel‐strike when measured quantitatively (i.e., foot contact patterns and hindlimb joint excursions) has been shown to vary in execution within and between hominoid taxa (Vereecke et al. 2003; Crompton et al. 2008; Zeininger et al. 2020) and is thus not a binary trait. Regardless of the degree of foot inversion and toe flexion observed during heel‐strike in orangutans, ultimately, the mechanism appears to be the same across the great apes, who are large‐bodied and must accomplish terrestrial locomotion with relatively longer forelimbs than hindlimbs. As noted by Zeininger et al. (2020), this morphology promotes increased hip translation and an extended knee during swing phase that brings the heel in contact with the ground first.

However, for the two orangutans who were observed walking bipedally with visible foot contact, no heel‐strike occurred (see Videos S2 and subadult in S11). This contrasts with observations made of orangutans walking bipedally in captive settings (Meldrum 1993; Crompton et al. 2003, 2008). The individual who took two steps, a flanged adult male, did so as he pulled down a sapling overhead to better access its leaves (Video S2). As such, the steps were slow and constrained in stride length, which may have affected foot placement. Future study is needed to better inform our understanding of the nature of foot contact in orangutans, given the probable variation in this feature. Additionally, the circumstances associated with this variation will be important to elucidate.

### 4.5 Relevance to the Origins of Hominin Bipedalism

Observations in this study demonstrate the maintained use of extended hip and knee joint positions on the ground in the absence of substrate compliance. While the consistency of arboreal and terrestrial joint excursions during orthograde behaviors offers some support for hypotheses for arboreal origins for hominin bipedalism (Hunt 1994; Hunt 1996; Crompton et al. 2003, 2008; Thorpe et al. 2014), the likelihood of an orangutan‐like *Pan‐Homo* LCA ultimately remains low given the degree of orangutan postcranial specialization. We suspect the anatomical complexes that confer exceptional joint flexibility in orangutans, affording full extension of the hip and knee, likely lead to joint instability, posing a challenge for terrestrial weight bearing. Specialized morphological features associated with orangutan hindlimb mobility include the absence of the ligamentum teres femoris in adults (Muchlinski et al. 2018), a particularly shallow acetabulum in the hip (MacLatchy and Bossert 1996), and a relatively convex articular surface to the proximal tibia in the knee (Zihlman et al. 2011; Zihlman and Underwood 2019). Although this study presents evidence for orangutans supporting their body weight terrestrially on one or two extended legs during mono‐ and bipedal stands, respectively, these postures were typically seen with the other limbs on surrounding supports, and in the case of the hindlimb, in highly abducted positions (see Videos S8 and S9). While captive orangutans have been noted for exhibiting full extension of the hindlimb on the ground (Tuttle and Cortright 1988; Crompton et al. 2003, 2008), our observations in the wild reveal an intrinsic tendency to distribute nonstanding limbs on nearby supports, underscoring their arboreal adaptation.

### 4.6 Terrestrial Orangutan Positional Behavioral Variability and Asymmetry

When comparing across the great apes, orangutans appear to demonstrate a relatively high level of positional variability when terrestrial, both within and between individuals. While variation should be expected, orangutans stand apart from the African apes in the apparent high degree of independence in the placement of all four limbs both in posture and locomotion. As a group, apes are recognized among primates to employ versatile positional behavior, both arboreally and terrestrially (Nowak and Reichard 2016), engaging in differential use of the fore‐ and hindlimbs (Young et al. 2010; MacLatchy et al. 2023). However, due to their distinct quadrumanous form of arboreality, the degree of inter‐ and intraindividual variability observed in orangutan terrestrial posture and locomotion may reflect decoupling movement not only between fore‐ and hindlimbs but between the lateral sides as well.

The apparent “sloppiness” of orangutan terrestrial locomotion has been recognized as long as they have been studied. Theodore Huxley (1894, 51) states “on the ground the Orang always goes laboriously and shakily on all fours” and further cites an 1841 quote from Sir James Brooke: “their motions are surprisingly awkward and uncouth”. The seemingly haphazard manner in which orangutans place their limbs indicates a lack of stereotypy in orangutan terrestrial positional behavior, with each movement determined by its specific context, as has been written of their behavior in the trees (Povinelli and Cant 1995).

The independence and plasticity of limb use is reflected in several findings of this study. First, hand and foot positions were found to be highly idiosyncratic. The variability of hand, wrist, and foot postures during quadrupedal locomotion as well as the asymmetry in posture between the hands and wrists within a locomotor bout suggest a pronounced adaptability of limb posture and movement within and between individuals. Second, overstride was found to be mixed as often as consistent in pattern during quadrupedal walking, indicating that leg dominance may be weak. Finally, the predominant use of monopedal over bipedal stands in this sample further supports lateral limb decoupling in orangutans. Quadrumanous thus remains an apt description of orangutan positional behavior, not only in the trees, but on the ground as well.

### 4.7 Summary

Orangutan terrestrial positional behavior has represented a gap in the hominoid literature, limiting comparisons across the great apes as well as knowledge of the full behavioral repertoire of *Pongo pygmaeus* . The results from this study have offered several new findings that not only expand our knowledge of great ape positional behavior but also inform longstanding debates and ambiguities concerning orangutan terrestriality in the wild.

Overall, the observations of terrestrial positional behavior of the wild orangutans in this study demonstrate substantial asymmetry, irregularity, and variation both within and between individuals. Limb placement and postures of the hands and feet appear to occur somewhat independent of one another, reflecting their adaptable behavior in the trees. This may suggest decoupling of not only the fore‐ and hindlimbs, as seen in other apes, but between the lateral sides as well.

## Author Contributions

**Emily R. Orlikoff:** investigation, methodology, writing – original draft, visualization, formal analysis, writing – review and editing, conceptualization, data curation, validation. **Gene R. Estrada:** visualization, writing – original draft, writing – review and editing. **Andrew J. Marshall:** data curation, funding acquisition, writing – review and editing. **Heiko U. Wittmer:** data curation, funding acquisition. **Endro Setiawan:** data curation, funding acquisition. **Cheryl D. Knott:** writing – review and editing, validation, data curation. **Lauren Sarringhaus:** conceptualization, investigation, writing – original draft, writing – review and editing, validation, project administration, supervision, methodology. **Laura M. MacLatchy:** writing – original draft, supervision, validation, writing – review and editing, conceptualization, project administration.

## Funding

This work was supported by Leakey Foundation; Victoria University of Wellington; University of Michigan; Association of Zoos and Aquariums (AZA) Ape Taxon Adivsory Group (TAG) Initiative; Mohamed bin Zayed Species Conservation Fund; Disney Conservation Fund.

## Conflicts of Interest

The authors declare no conflicts of interest.

## Supporting information


**Video S1:** The adult female, Batang, at the Smithsonian Zoo knuckle‐walks on a wet floor, supporting previous observations in captivity.

**Video S2:** Extended bipedal walk performed by a flanged adult male. Note the lack of heel‐strike in these steps.

**Video S3:** Crutch walk performed by an adult of unknown sex.

**Video S4:** Extended bipedal stand performed by an adult male. He walks quadrupedally and then stands with fully extended hindlimbs. He places his right hand on a tree for balance while standing, but the forelimb does not bear significant weight.

**Video S5:** Extended monopedal stand performed by an adult female. She orthograde stands with her left hindlimb fully extended. She initially holds a horizontal branch with her right hand and then also grabs the same branch with her right foot. Given the modest rebound of the horizontal branch when first grabbing it with her foot, it is unlikely that she is placing substantial weight on the branch once she stabilizes.

**Video S6:** Bipedal stand performed by an adult female. She walks quadrupedally, pauses and orthograde stands with fully extended hindlimbs (no hand assistance), then continues quadrupedal walking. She is followed by an adult male quadrupedal walking.

**Video S7:** Extended monopedal stand/forelimb suspend performed by an adult female. She walks quadrupedally, then stands on her left leg while grabbing a small tree with her right forelimb high in suspension. She then uses her left foot and right hand to pull a branch toward her to feed.

**Video S8:** Forelimb‐hindlimb swing performed by an adult female. She stands monopedally with a fully extended hindlimb, using the forelimb in suspension. She then swings around the tree by her right forelimb and hindlimb and stands monopedally again.

**Video S9:** Extended monopedal stand with forelimb compression performed by an adult male.

**Video S10:** Quadrupedal walk performed by an adult female with toes flexed and directed medially and the foot oriented mostly anteroposteriorly.

**Video S11:** Quadrupedal walk performed by an adult female with the hallux extended medially and the remaining digits flexed and directed laterally. A subadult can also be seen in a flexed bipedal stand with forelimb assistance using the trunk of the tree. The subadult then takes a single hand‐assisted, flexed bipedal step without heel‐strike and transitions to quadrupedal walking.

## Data Availability

The data that support the findings of this study are openly available in Figshare at https://doi.org/10.6084/m9.figshare.31286314.
